# Supplementary figures and images for: AMiGA: Software for Automated Analysis of Microbial Growth Assays
Source: mSystems. 2021 Jul 13;6(4):e00508-21. doi: 10.1128/mSystems.00508-21 (PMC8409736; doi:10.1128/mSystems.00508-21)

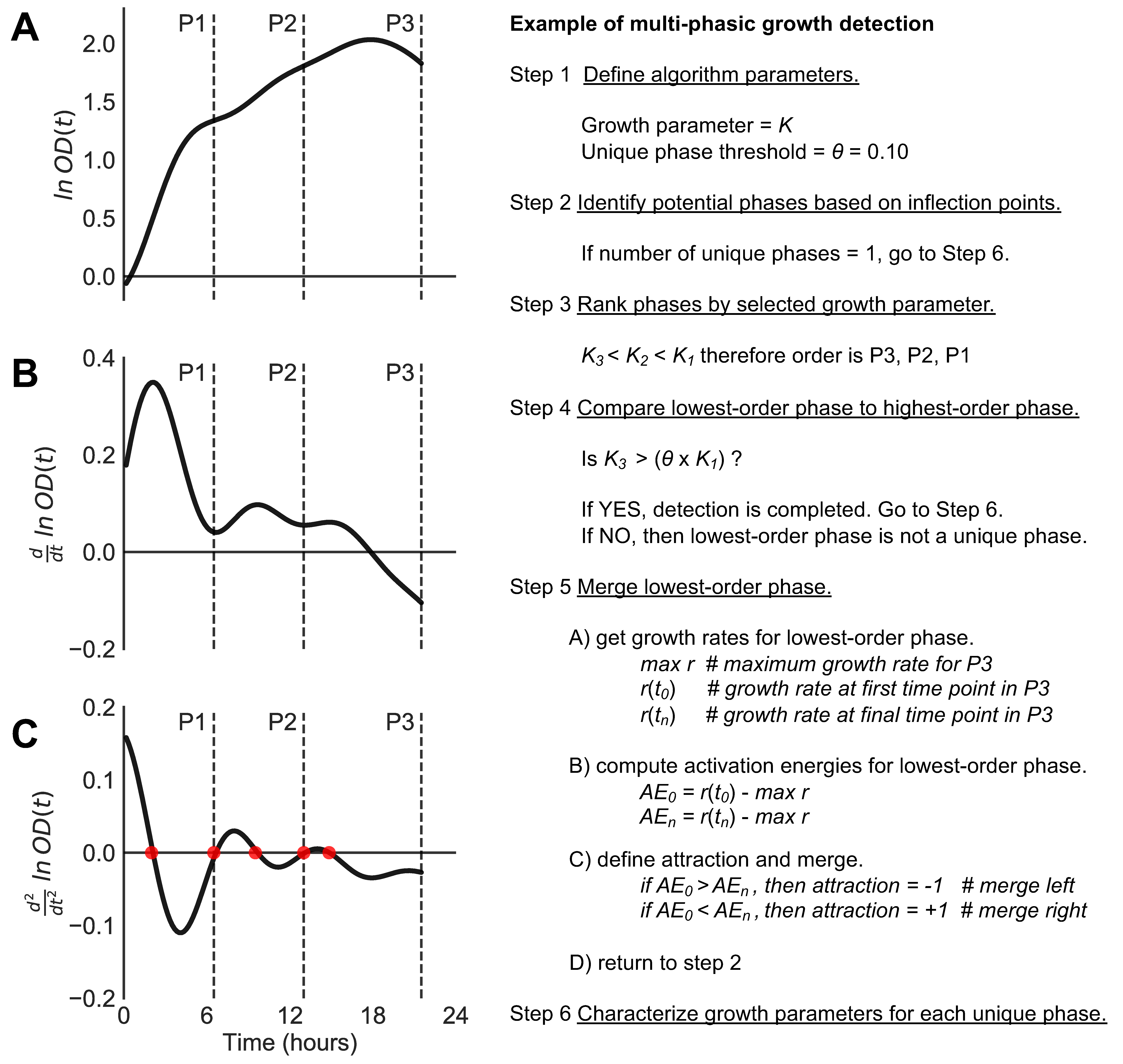

Supplement: FIG S1 [file msystems.00508-21-sf001.tif]

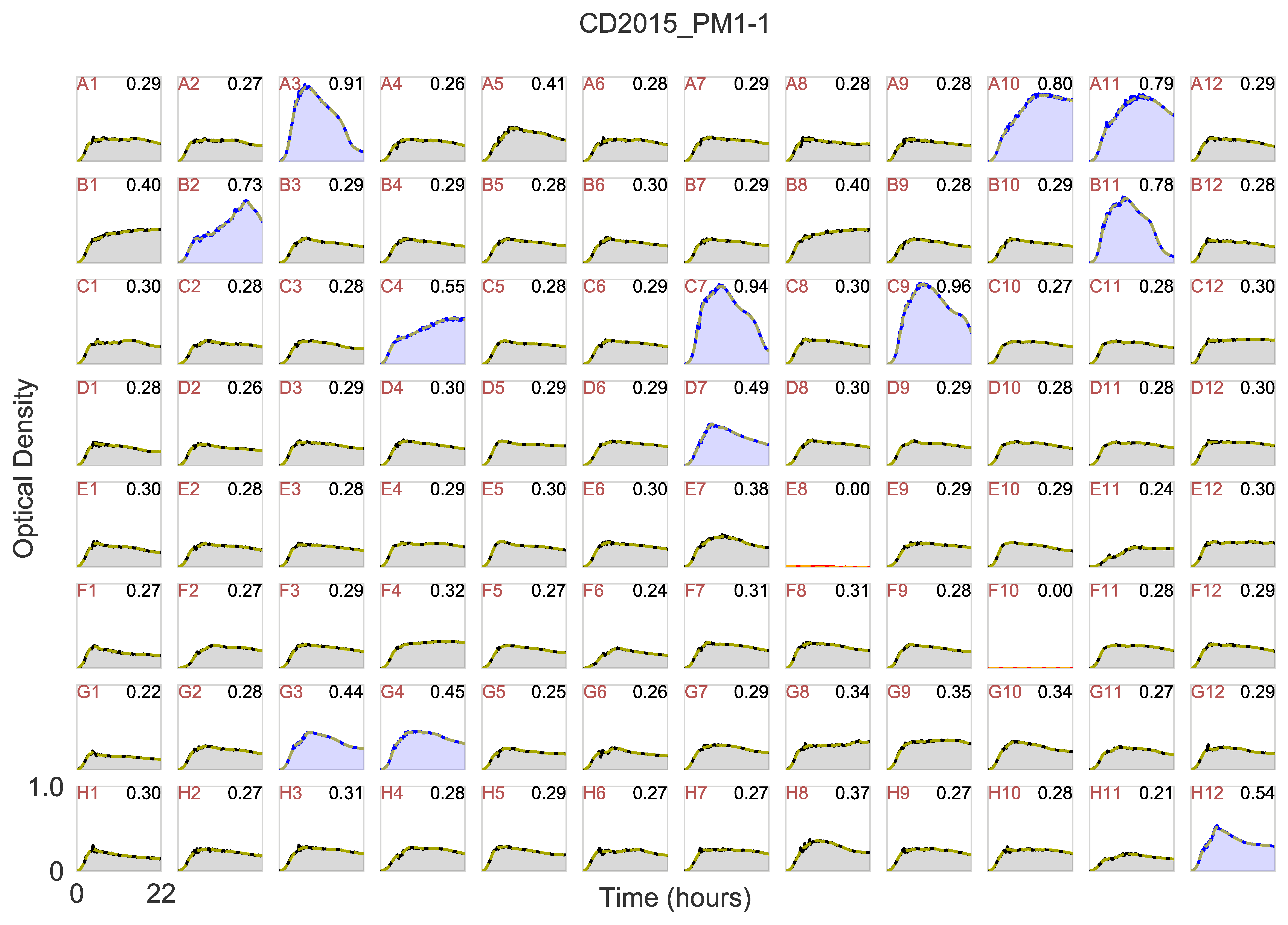

Supplement: FIG S2 [file msystems.00508-21-sf002.tif]

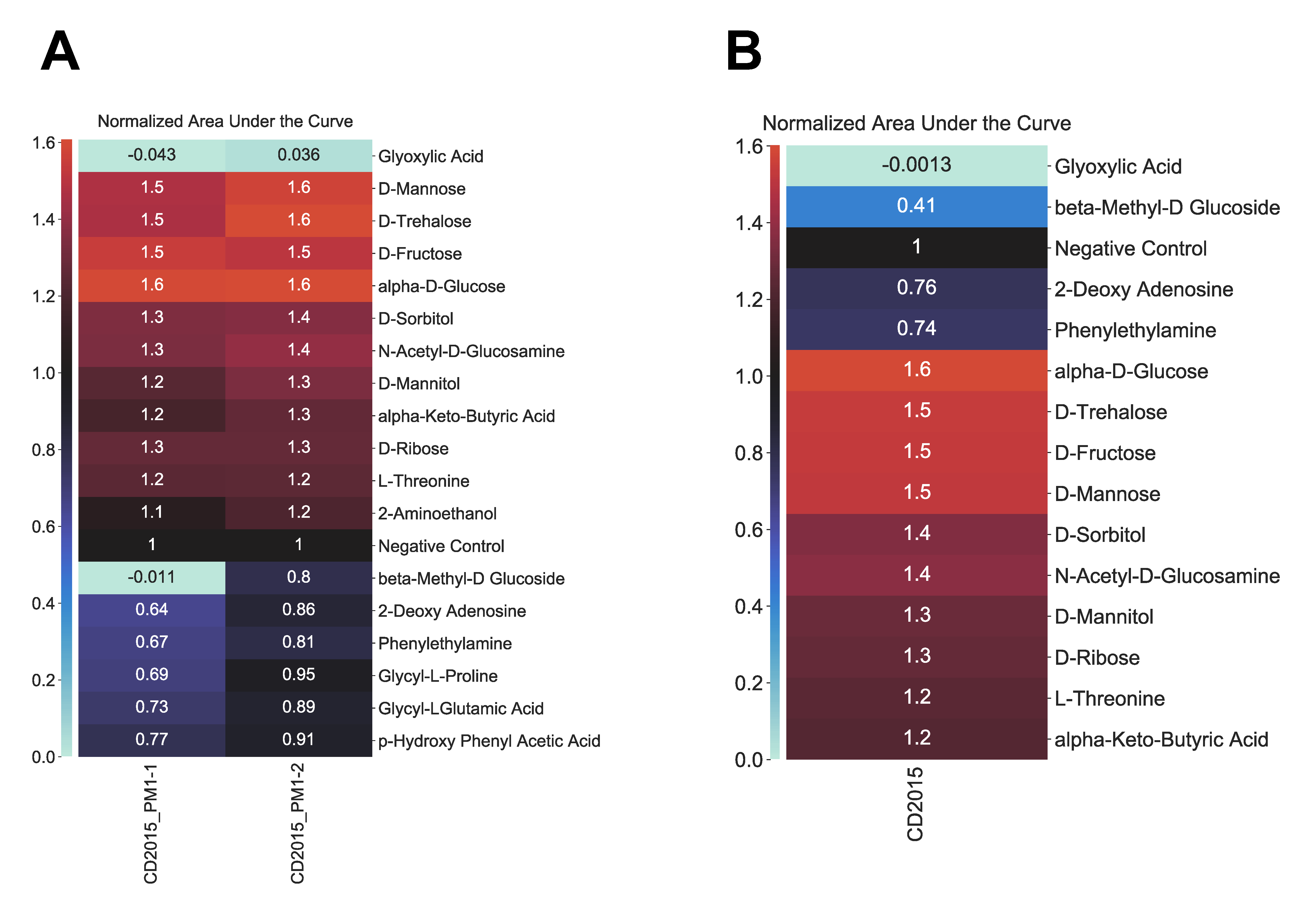

Supplement: FIG S3 [file msystems.00508-21-sf003.tif]

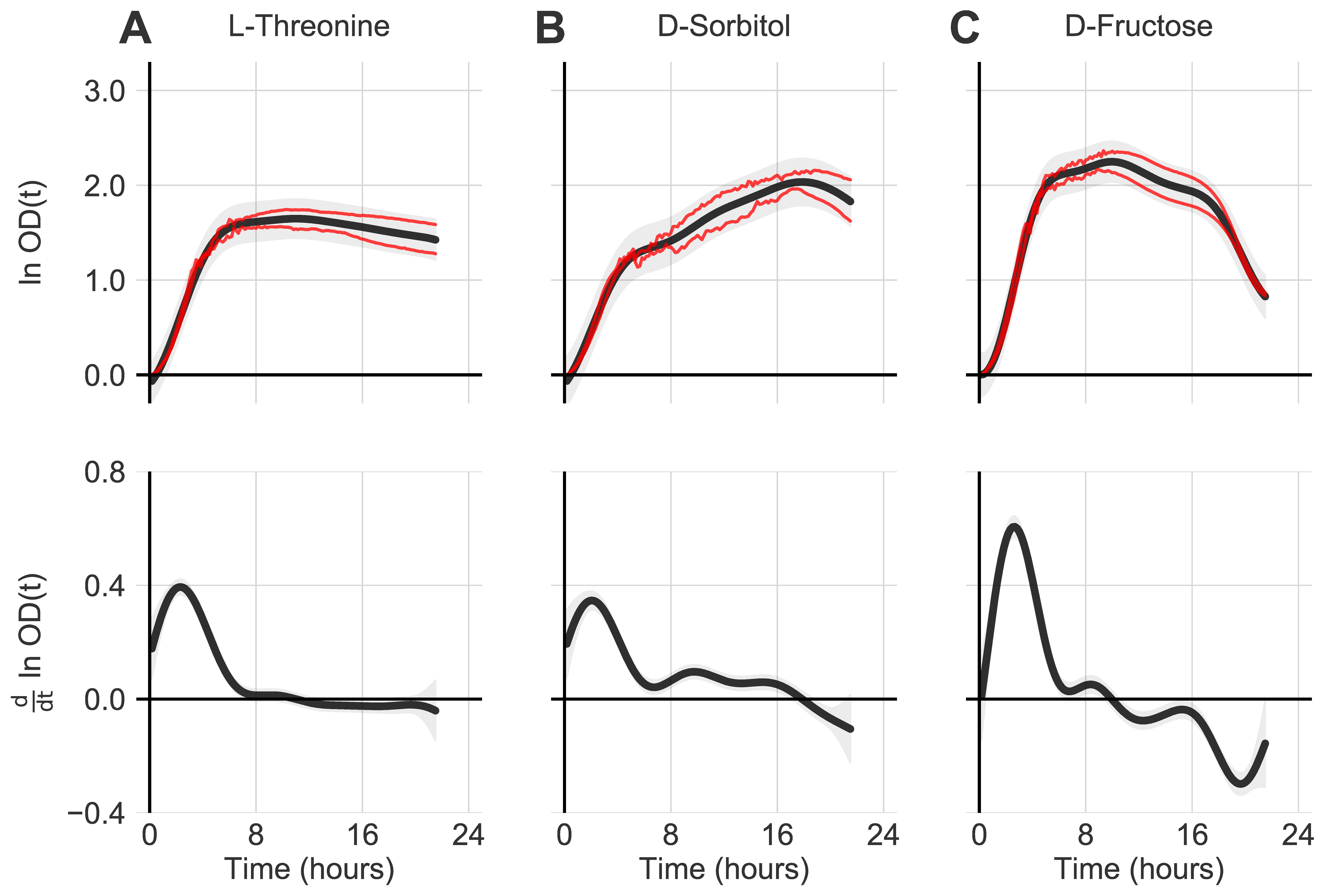

Supplement: FIG S4 [file msystems.00508-21-sf004.tif]

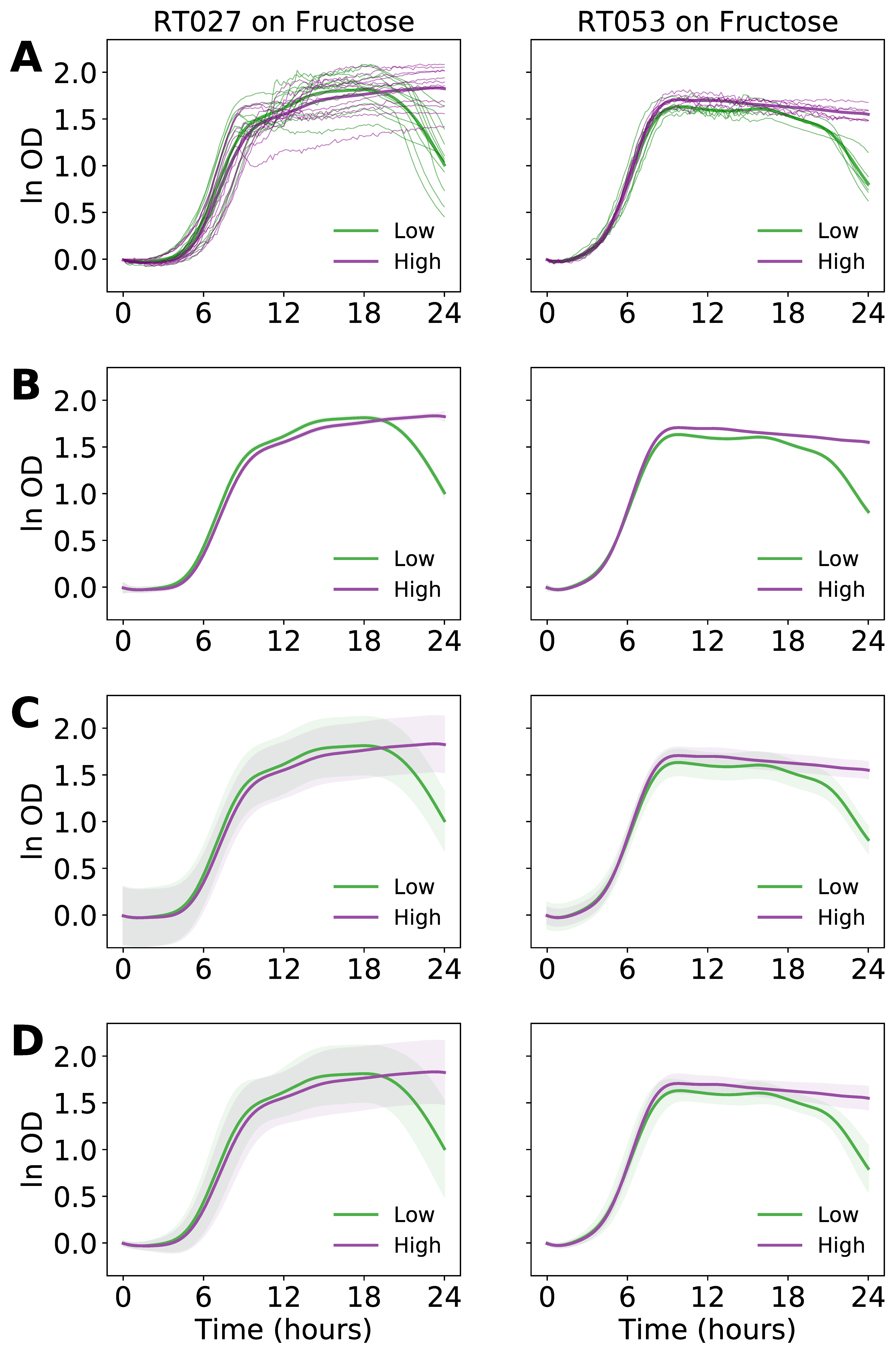

Supplement: FIG S6 [file msystems.00508-21-sf006.tif]
