# Supplementary material for: AMiGA: Software for Automated Analysis of Microbial Growth Assays
Source: mSystems. 2021 Jul 13;6(4):e00508-21. doi: 10.1128/mSystems.00508-21 (PMC8409736; doi:10.1128/mSystems.00508-21)
Supplement: TABLE S1 [file msystems.00508-21-st001.docx]

**Supplemental Table 1**

| **Strain** | **Toxin type** | **PFGE type**  **(NAP status)** | **Ribotype** |
| --- | --- | --- | --- |
| CD2015 | III | MI-NAP1 | RT027 |
| CD4015 | III | MI-NAP1 | RT027 |
| CD4001 | III | MI-NA13 | RT027 |
| CD4010 | III | MI-UN13 | RT027 |
| CD1015 | V | MI-NAP7 | RT078 |
| M120 | V | NA | RT078 |
| CD2001 | V | MI-NAP8 | RT078 |
| CD3014 | 0 | MI-NAP2 | RT001 |
| CD4011 | 0 | MI-NAP2 | RT001 |
| CD1007 | 0 | MI-NAP3 | RT053 |
| CD2048 | 0 | MI-NAP3 | RT053 |
| CD2058 | NA | MI-UN5 | NA |
