# Supplementary material for: AMiGA: Software for Automated Analysis of Microbial Growth Assays
Source: mSystems. 2021 Jul 13;6(4):e00508-21. doi: 10.1128/mSystems.00508-21 (PMC8409736; doi:10.1128/mSystems.00508-21)
Supplement: TABLE S2 [file msystems.00508-21-st002.docx]

**Supplemental Table 2**

| **Concentration** | **Low** | **High** | **Low** | **High** |  |
| --- | --- | --- | --- | --- | --- |
| **Parameter** | **Mean** | **Mean** | **95.0% CI** | **95.0% CI** | **Sig. Diff.** |
| AUC (log) | 27.154 | 29.891 | [27.026,27.282] | [29.805,29.976] | TRUE |
| Diauxie | 0 | 0 | NA | NA | FALSE |
| Death (log) | 0.829 | 0.153 | [0.785,0.874] | [0.119,0.188] | TRUE |
| Death Rate | -0.23 | -0.04 | [-0.267,-0.193] | [-0.083,0.003] | TRUE |
| Growth Rate | 0.395 | 0.429 | [0.379,0.411] | [0.419,0.440] | TRUE |
| Carrying Capacity (log) | 1.635 | 1.708 | [1.618,1.652] | [1.697,1.719] | TRUE |
| Lag Time | 3.98 | 4.085 | [3.876,4.083] | [4.025,4.146] | FALSE |
| Adaptation Time | 0.202 | 0.347 | [-0.422,0.825] | [-0.388,1.082] | FALSE |
| Time at Max. Death Rate | 23.17 | 21.213 | [22.327,24.013] | [14.038,28.388] | FALSE |
| Time at Max. Growth Rate | 6.238 | 6.24 | [6.069,6.407] | [6.077,6.403] | FALSE |
| Time at Carrying Capacity | 10.077 | 10.21 | [7.781,12.372] | [8.392,12.028] | FALSE |
| Doubling Time | 1.755 | 1.614 | [1.684,1.826] | [1.576,1.652] | TRUE |
